# Dual-encoder 3D transformer-based U-Net with temporal attention for non-mass enhancement segmentation in breast dynamic contrast-enhanced MRI

**DOI:** 10.1007/s12194-025-01004-y

**Published:** 2026-01-08

**Authors:** Tomoki Kosugi, Ryohei Nakayama, Koji Sakai, Mariko Goto

**Affiliations:** 1 Graduate School of Science and Engineering, Ritsumeikan University, 1-1-1 Noji- higashi, Kusatsu, 525-8577 Shiga Japan; 2 Graduate School of Medical Sciences, Department of Radiology, Kyoto Prefectural University of Medicine, 465 Kajii-cho, Kawaramachi-Hirokoji, Kamigyo- ku, Kyoto, 602-8566 Japan

**Keywords:** Non-mass enhancement, Breast DCE-MRI, 3D transunet, Temporal attention, Medical image segmentation

## Abstract

Non-mass enhancement (NME) in breast dynamic contrast-enhanced magnetic resonance imaging (DCE-MRI) often shows subtle and diffuse enhancement, which can make accurate delineation challenging for radiologists. In this study, we developed an automated segmentation method for NME in DCE-MRI using a three-dimensional (3D) TransUNet model with a temporal attention mechanism. The attention module uses images of the difference between the pre- and early post-contrast phases to highlight regions that exhibit enhancement-related temporal changes. Our database consisted of 151 DCE-MRI cases with NME, each including both pre- and early post-contrast phases. The proposed network comprises two parallel CNN encoders (one for early post-contrast images and one for early–pre subtraction images), a temporal attention module, a transformer encoder, and a CNN decoder. The two encoders independently extract features from their respective inputs. The temporal attention module uses the features from the difference branch to weight those of the early branch. The weighted features are then fused and passed to the transformer encoder, which captures spatial and contextual dependencies within the 3D volume. The CNN decoder reconstructs the NME segmentation map through transposed convolutions and upsampling layers. Using five-fold cross-validation, the proposed method achieved a Jaccard index of 0.724 and a Dice coefficient of 0.802. Our approach significantly outperformed the baseline 3D TransUNet (Jaccard: 0.585, *p* < .001; Dice: 0.690, *p* < .001). These results demonstrate the high segmentation performance of the proposed method for NME and indicate its potential to improve diagnostic assessment in breast DCE-MRI.

**Supplementary Information:**

The online version contains supplementary material available at 10.1007/s12194-025-01004-y.

##  Introduction

Breast cancer is the most common cancer among women, accounting for approximately 670,000 deaths worldwide in 2022 [1]. Among various imaging modalities, dynamic contrast-enhanced magnetic resonance imaging (DCE-MRI) is widely used for the clinical assessment of breast lesions and for delineating the extent of disease involvement. DCE-MRI provides critical temporal information on contrast enhancement, which facilitates the evaluation of tumor vascularity and lesion dynamics. However, a subtype known as non-mass enhancement (NME) poses a considerable diagnostic challenge in DCE-MRI. NME often presents subtle and diffuse contrast uptake without forming a well-defined mass, which makes it difficult for even experienced radiologists to accurately delineate its extent [2–4]. Accurate segmentation of NME is clinically important because it may indicate the presence of ductal carcinoma in situ (DCIS) or other malignant processes [5]. Therefore, the development of automated and reliable segmentation methods for NME is highly desirable to support diagnostic interpretation and reduce inter-observer variability [6].

Deep learning has achieved remarkable success in medical image segmentation, with the U-Net architecture being among the most widely adopted approaches [7–10]. U-Net is a convolutional neural network (CNN) based on an encoder-decoder architecture with skip connections between corresponding resolution levels to allow the decoder to recover fine-grained spatial information [11]. While effective at capturing local features, U-Net exhibits limited capacity to capture long-range dependencies due to its inherently localized convolutional operations. To overcome this limitation, vision transformer (ViT)-based models have been introduced in the field of medical imaging [12–17]. ViT divides input images into patches and applies self-attention mechanisms to capture inter-patch relationships to model long-range dependencies and global context [18, 19]. However, ViT does not inherently incorporate the inductive biases found in convolutional networks, such as spatial locality and translation invariance. These characteristics are essential for identifying fine-grained anatomical boundaries.

Hybrid architectures such as 3D TransUNet have been proposed to combine the advantages of convolutional networks and transformer models [20]. 3D TransUNet combines the local feature extraction capabilities of convolutional encoders with the strong global context modeling of transformer modules to achieve promising performance in volumetric medical image segmentation. Nevertheless, segmentation of NME remains particularly difficult for such models owing to the lesion’s low conspicuity and heterogeneous morphologies in breast DCE-MRI [21].

To address this issue, we propose a novel segmentation method called 3D TransUNet with temporal attention that employs two parallel CNN encoders, with one for the early post-contrast images and one for the early–pre subtraction images. The temporal attention module uses the features extracted by the difference branch to weight those obtained by the early branch to emphasize enhancement-related temporal changes and improve the localization and delineation of NME regions in breast DCE-MRI. This design integrates convolutional inductive biases with the long-range context of a transformer model to enhance segmentation accuracy by leveraging subtle enhancement dynamics that static image features alone may miss.

## Materials and methods

###  Materials

This retrospective study was approved by the institutional review board of Kyoto Prefectural University of Medicine (approval number: ERB-C-1763). The requirement for informed consent was waived due to the use of anonymized imaging data.

DCE-MRI data were collected from 151 patients diagnosed with NME between February 2020 and November 2023 at Kyoto Prefectural University of Medicine Hospital (Kyoto, Japan). All examinations were performed using a 3.0-T MRI scanner (Magnetom Skyra; Siemens Healthcare, Erlangen, Germany) equipped with a 16-channel dedicated breast coil. Each case consisted of two imaging phases, including a pre-contrast phase and an early post-contrast phase. The early phase was acquired 90 s after intravenous administration of a gadolinium-based contrast agent. Both phases were acquired with identical imaging parameters, including an in-plane matrix size of 352 × 352 pixels, 144 axial slices, and a voxel size of 0.91 × 0.91 × 1.0 mm.

The NME regions were manually annotated on the early post-contrast images by a board-certified breast imaging radiologist with more than 17 years of experience in interpreting breast MRI. All annotations were performed using 3D Slicer (version 5.8.1; https://www.slicer.org). These annotations were used to generate ground-truth segmentation masks that served as a reference standard for training and evaluating the proposed model.

To minimize the influence of surrounding anatomical structures such as the thoracic cage, regions of interest (ROIs) including the breast area were extracted from the DCE-MRI images. Following the method described by Hizukuri et al. [22], the breast foreground was first segmented on the early post-contrast image using gray-level thresholding. The left and right nipples were then automatically detected as the most anterior breast points in each half of the image, and the midpoint between them was used as a superior landmark; an inferior landmark at the level of the sternum was subsequently identified along this midline. A rectangular breast region spanning the full left–right extent and the vertical range between these landmarks was extracted, and this region was centrally cropped to 176 × 176 pixels for both the pre- and early post-contrast images. Thus, the ROI was defined based on fixed anatomical landmarks rather than being centered on the annotated NME.

All the development, training, and evaluation procedures were implemented in Python and conducted on a workstation equipped with an Intel Core i7-12700 CPU, 128 GB of RAM, and an NVIDIA GeForce RTX 3060 GPU with 12 GB of VRAM.

### Generation of temporal difference images

Temporal contrast enhancement patterns are important as imaging features for diagnosing NME in breast DCE-MRI [23]. To enable the model to learn temporal characteristics, difference images were generated by subtracting the pre-contrast images from the early post-contrast images. To ensure accurate subtraction, rigid registration was applied to align the pre- and early post-contrast images [24]. This spatial alignment minimized the effects of patient motion and positional shifts that may have occurred between acquisitions. Following registration, difference images were determined on a voxel-wise basis by subtracting each voxel intensity in the pre-contrast image from the corresponding value in the aligned early post-contrast image. To emphasize regions with increased signal intensity due to contrast uptake, only positive values in the resulting difference images were preserved. Negative values, which are often attributed to noise or minor registration inaccuracies, were set to zero to suppress irrelevant signal fluctuations and enhance diagnostically meaningful features.

### 3D transunet with temporal attention

**Fig. 1:**
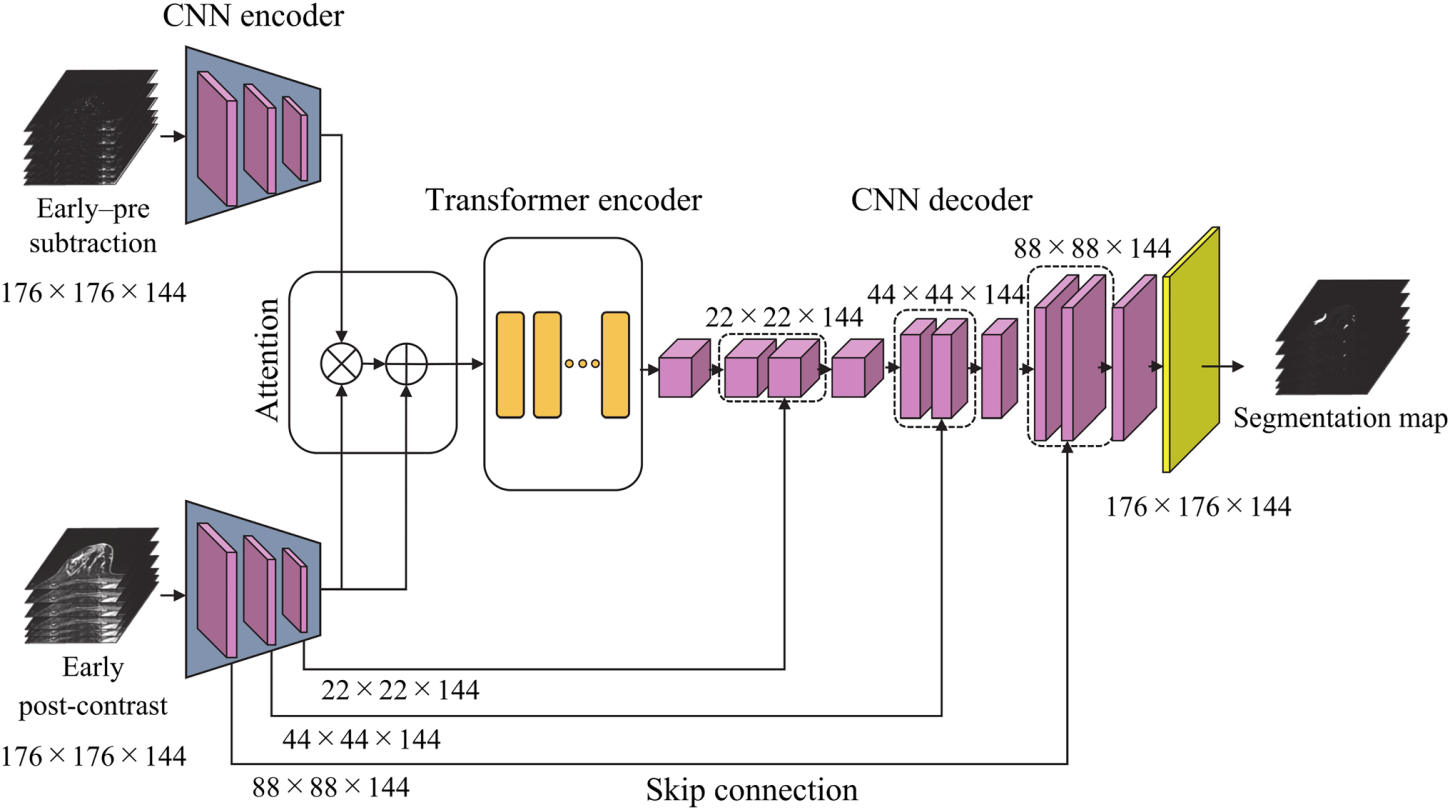
Overview of the 3D TransUNet with temporal attention

**Fig. 2:**
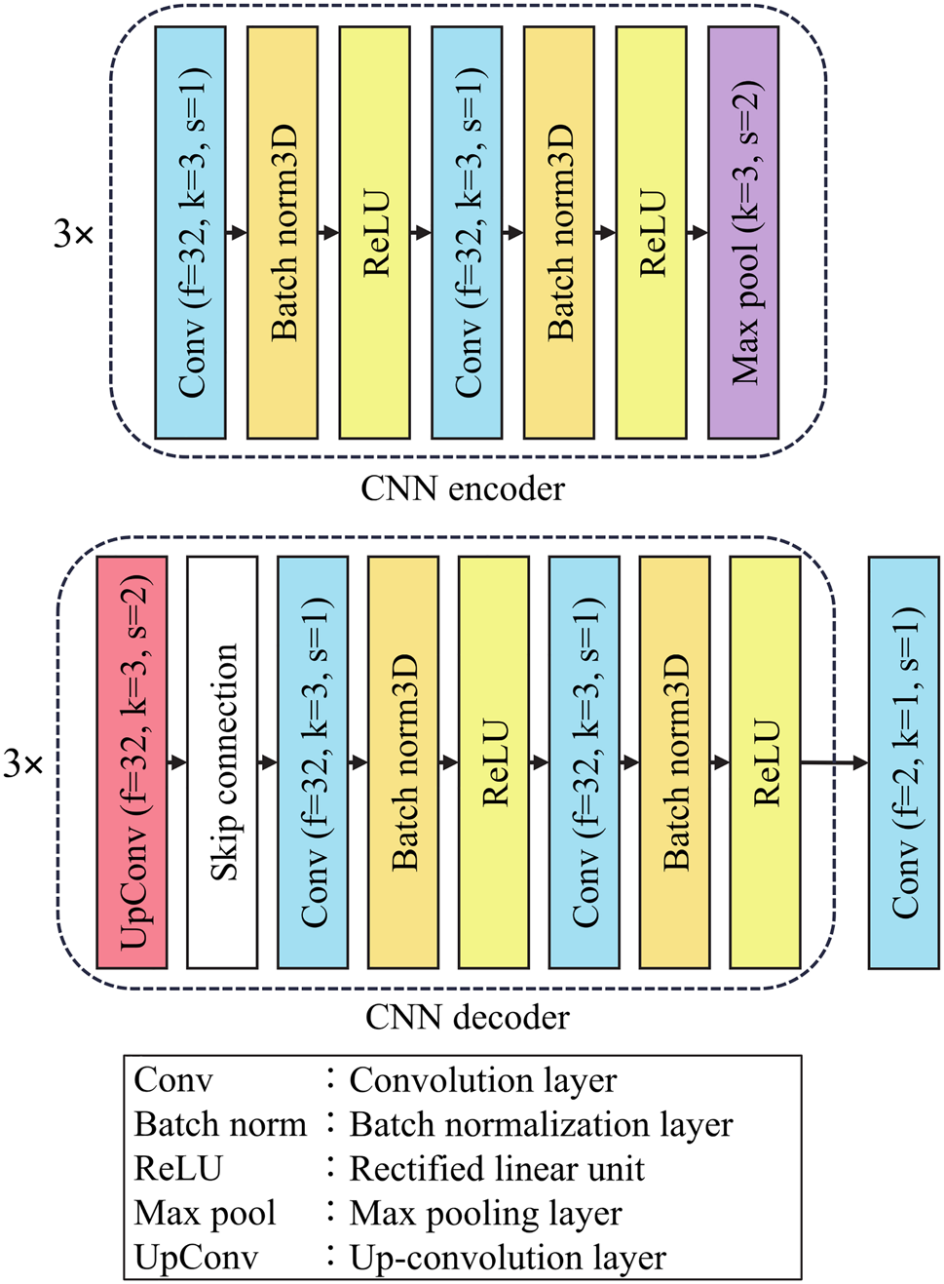
CNN encoder and decoder architectures

**Fig. 3:**
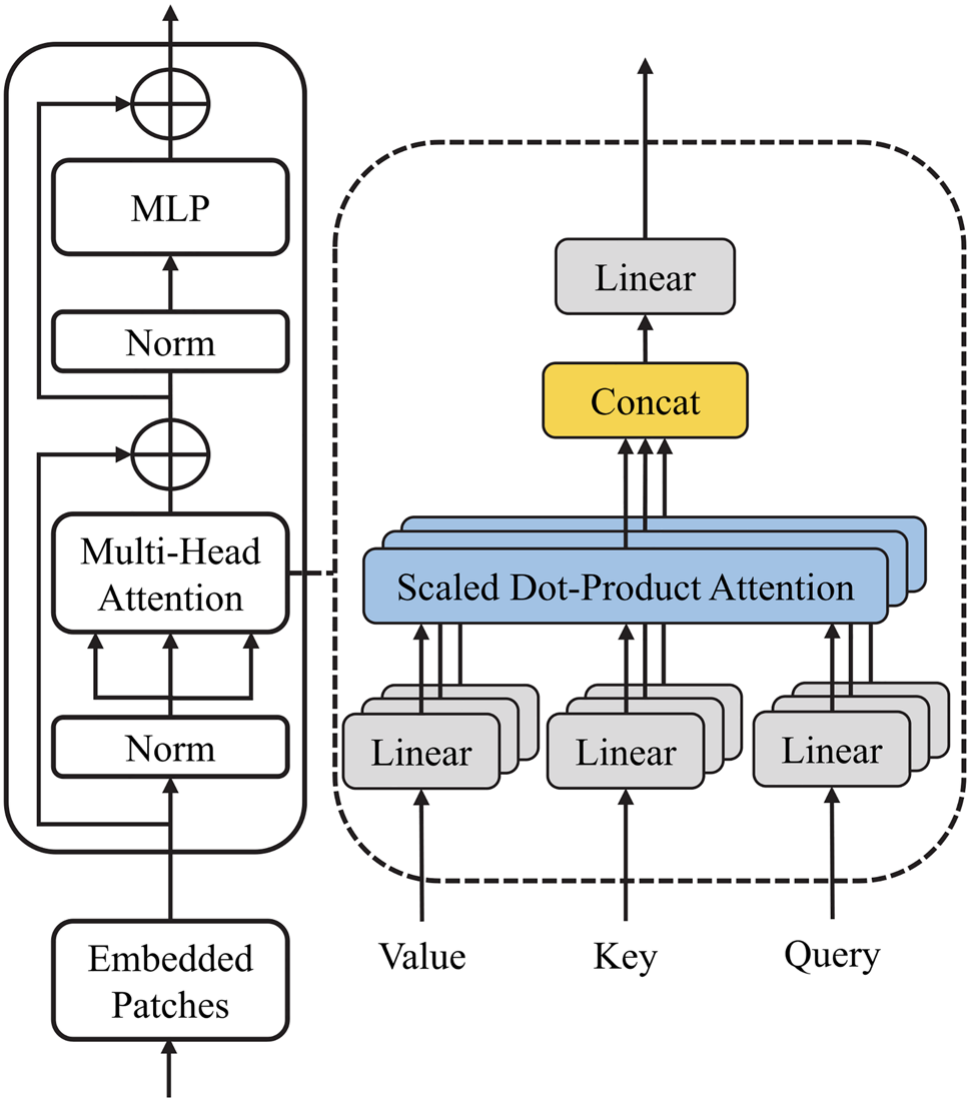
Architecture of the transformer encoder

The proposed network consists of four main components, including two parallel CNN encoders (one for the early post-contrast images and one for the early–pre subtraction images), a temporal attention mechanism, a transformer encoder, and a CNN-based decoder. An overview of the network architecture is shown in Fig. 1. Details of the CNN encoder and decoder are provided in Fig. 2, and the internal structure of the transformer encoder is illustrated in Fig. 3.

The proposed model takes a pair of 3D volumes with a size of 176 × 176 × 144 voxels as input, which are the early post-contrast image and the image created as the difference between the pre-contrast and early post-contrast images. These two inputs are processed independently through the two CNN encoders, each of which includes three sequential feature extraction blocks. Each block comprises a 3D convolutional layer, batch normalization, a rectified linear unit (ReLU) activation function, and a max-pooling layer. After encoding, both inputs are transformed into feature maps with a size of 22 × 22 × 144 pixels.

To generate the temporal attention map, an element-wise product is determined between the two encoded feature maps. This operation highlights regions that exhibit significant temporal changes in signal intensity. Let $\:{F}_{\mathrm{early}}\in\:{\mathbb{R}}^{C\times\:22\times\:22\times\:144}$ and $\:{F}_{\mathrm{diff}}\in\:{\mathbb{R}}^{C\times\:22\times\:22\times\:144}$ denote the feature maps obtained from the early post-contrast image and the early–pre subtraction image, respectively. The proposed difference-driven attention output is defined as

1 $$ \:F_{{{\mathrm{att}}}} = F_{{{\mathrm{early}}}} + F_{{{\mathrm{early}}}} \odot \:F_{{{\mathrm{diff}}}} \:, $$

where $\:\odot\:$ denotes element-wise multiplication. In this equation, voxels where both $\:{F}_{\mathrm{early}}$ and $\:{F}_{\mathrm{diff}}$ have large magnitude (i.e., strong early enhancement and large temporal change) are multiplicatively amplified, whereas regions with small difference values are relatively suppressed. The residual addition term preserves the original early-phase representation and stabilizes optimization, while allowing the difference features to act as a gate that selectively enhances temporally active regions. The resulting attention map is then added element-wise to the early-phase feature map to produce an attention-enhanced representation with a size of 22 × 22 × 144 pixels. This representation emphasizes regions corresponding to contrast uptake.

The attention-enhanced feature map is then passed to the transformer encoder, which is composed of two layer-normalization operations, a multi-head self-attention module, and a multi-layer perceptron. The input feature map is divided into non-overlapping 3D patches of size 8 × 8 × 8. Each patch is vectorized and embedded with positional information to retain spatial context. The multi-head self-attention module applies scaled dot-product attention to model the relationships between patches to enable the network to capture long-range spatial dependencies and structural context throughout the 3D volume.

Finally, the CNN decoder based on the 3D U-Net architecture reconstructs the segmentation map from the encoded features. It comprises transposed convolutional layers for upsampling, followed by convolutional layers with batch normalization and ReLU activation. Skip connections from the encoder to the decoder are incorporated at corresponding levels of resolution to preserve fine-grained spatial information. The final output is a 3D segmentation map that retains the same spatial dimensions as the original input image.

### Model training

The proposed 3D TransUNet with temporal attention was trained using the Dice loss, which is widely adopted in medical image segmentation owing to its effectiveness in addressing class imbalance. The Dice loss is defined as follows.

2 $$ \:\begin{array}{*{20}c} {Dice\:loss = 1 - \frac{{2\sum \: _{{i = 1}}^{n} p_{i} q_{i} }}{{\sum \: _{{i = 1}}^{n} (p_{{i + }} q_{i} )}},} \\ \end{array} $$

where $\:p$ denotes the ground-truth label and $\:q$ represents the predicted probability map generated by the network. This loss function directly optimizes the spatial overlap between the predicted and ground-truth segmentation regions, which makes it particularly suitable for segmentation tasks involving sparse or small target regions. The model was trained with a batch size of 2 for up to 100 epochs. The AdamW optimizer was employed with a learning rate of 1 × 10⁻⁴. To improve convergence and generalization, the transformer encoder was initialized with pre-trained weights from ImageNet-21k [25].

To evaluate the robustness of the model’s performance, a five-fold cross-validation was conducted using patient-level grouping ($\:k=5$) [26]. The dataset comprising ROIs for 151 patients was randomly divided into five mutually exclusive folds to ensure that each fold contained approximately the same number of patients. One fold was used as the testing dataset and the remaining four were used for training. This process was repeated five times such that each fold served as the testing dataset exactly once.

### Evaluation metrics

The evaluation was conducted using five-fold cross-validation, with the results reported as averages across the test folds. We used the Jaccard index and the Dice coefficient as two standard overlap-based measures to assess segmentation accuracy. They are defined as follows.

3 $$ \:\begin{array}{*{20}c} {Jaccard\:index = \frac{{|A \cap \:B|}}{{|A \cup \:B|}},} \\ \end{array} $$

4 $$ \:\begin{array}{*{20}c} {Dice\:coefficient = \frac{{2|A \cap \:B|}}{{\left| A \right| + \left| B \right|}},} \\ \end{array} $$

where $\:A$ represents the predicted NME region and B denotes the corresponding ground truth mask.

### Comparison study design

The performance of the proposed dual-encoder 3D TransUNet with difference-driven temporal attention was quantitatively evaluated and compared with three variants, while keeping the transformer encoder and CNN decoder identical across all models. (i) Baseline (standard 3D TransUNet): a conventional 3D TransUNet with a single CNN encoder receiving a three-channel input (pre-contrast, early post-contrast, and early–pre subtraction image) and no temporal attention mechanism. (ii) Single-Encoder + Attention: a single CNN encoder also received the same three-channel input; its output feature map was used to construct Query/Key/Value for the attention operation, and the attended features were then forwarded to the transformer encoder. (iii) Dual-Encoder without Attention: two parallel CNN encoders independently processed (a) the early post-contrast image and (b) the early–pre subtraction image; the resulting feature maps were concatenated and fed into the shared transformer encoder and CNN decoder without an attention module. (iv) Proposed: identical to (iii), except that the concatenated features were passed through the temporal attention module before the shared transformer encoder and CNN decoder.

In addition, the proposed model was evaluated against two recent Transformer-based hybrid segmentation models, Swin-UNETR [27] and nnFormer [28]. For a fair comparison, both Swin-UNETR and nnFormer were trained and tested using the same three-channel input as the baseline model (pre-contrast, early post-contrast, and early–pre subtraction image), concatenated along the channel dimension and fed into each network.

The Wilcoxon signed-rank test was applied to determine whether the observed performance differences between the proposed model and the other 3D TransUNet variants (baseline, Single-Encoder + Attention, and Dual-Encoder without Attention) were statistically significant. This nonparametric test is appropriate for paired data that do not necessarily follow a normal distribution. Statistical analysis was conducted using the R software environment (version 4.5.1, R Foundation for Statistical Computing, Vienna, Austria), with the significance level set at *p* < .05. The same Wilcoxon signed-rank test was also used to compare the proposed model with Swin-UNETR and nnFormer. When multiple pairwise comparisons were conducted, p-values were adjusted using the Holm method to control the family-wise error rate.

## Results

**Fig. 4:**
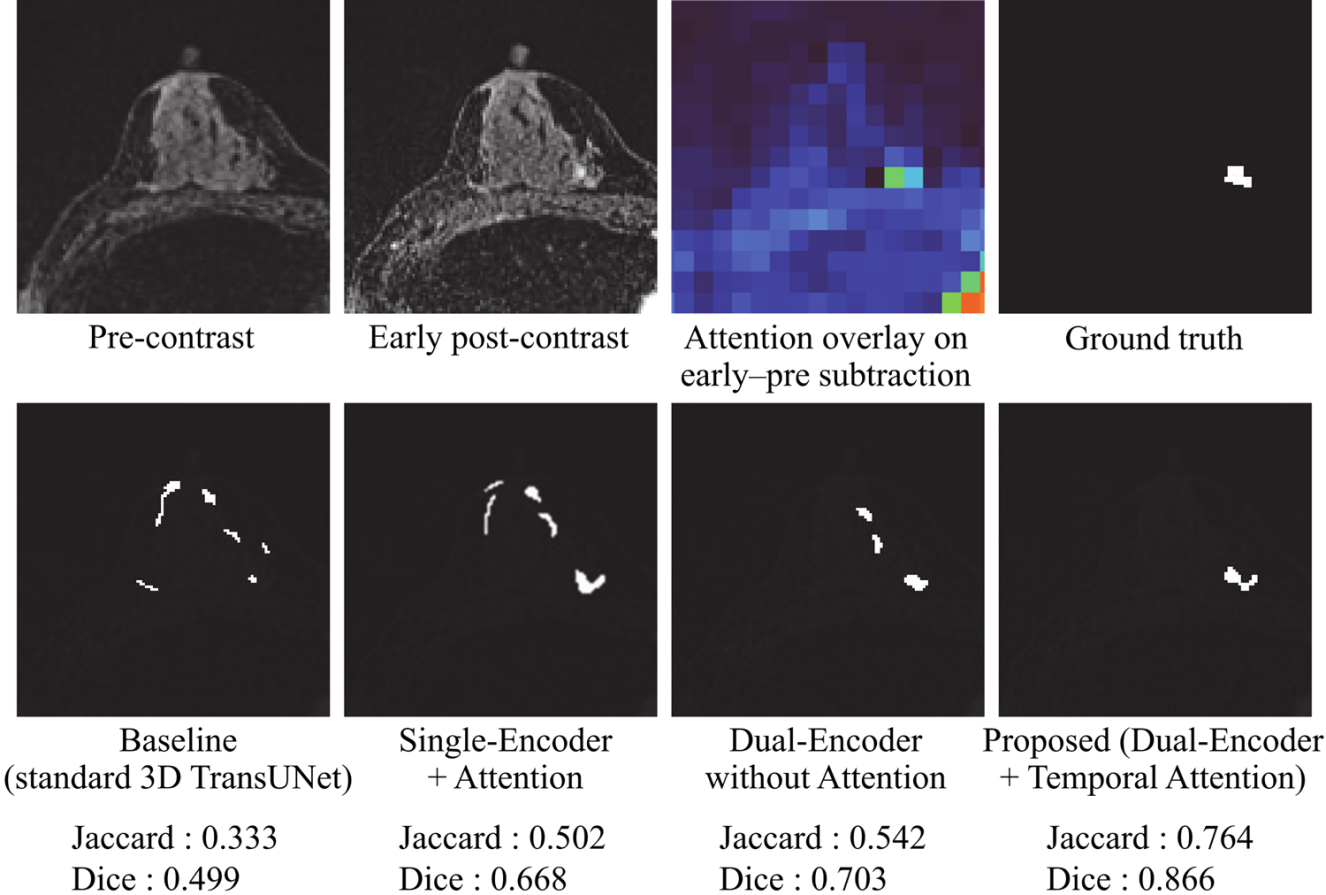
Representative segmentation results for the four ablation models: (i) Baseline, (ii) Single-Encoder + Attention, (iii) Dual-Encoder without Attention, and (iv) Proposed (Dual-Encoder + Temporal Attention). The top row shows the input images, the early–pre subtraction image with the attention map overlaid as a heatmap, and the ground truth mask; the bottom row shows the predicted masks with case-wise Jaccard and Dice

**Fig. 5:**
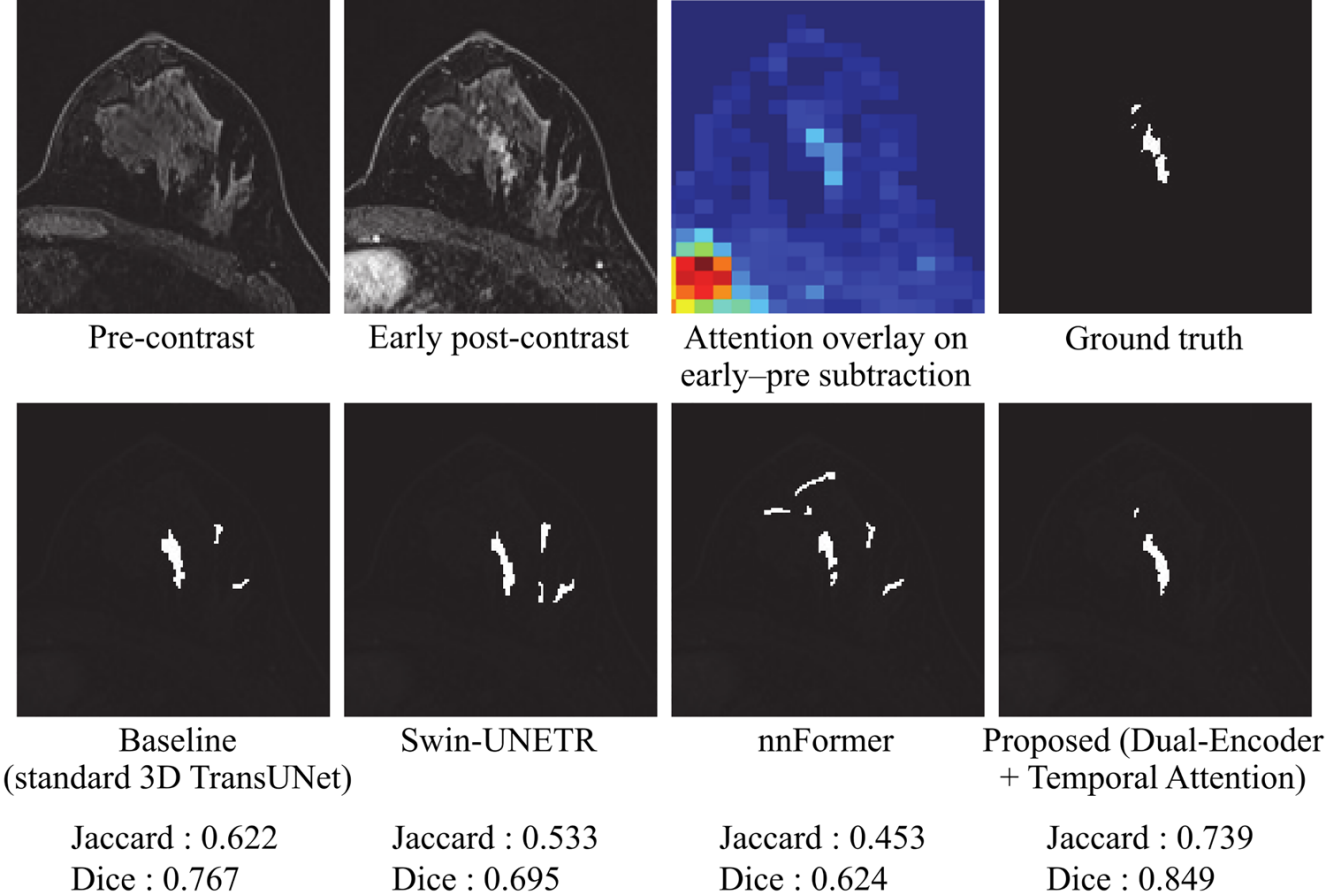
Representative segmentation results for the baseline 3D TransUNet, Swin-UNETR, nnFormer, and the proposed dual-encoder 3D TransUNet with difference-driven temporal attention

Figure 4 presents representative segmentation results for the four 3D TransUNet variants in the ablation study: the baseline 3D TransUNet, Single-Encoder + Attention, Dual-Encoder without Attention, and the proposed Dual-Encoder + Temporal Attention. The proposed model delineated NME boundaries more accurately and suppressed false positives in regions with high signal intensity, such as blood vessels and fibroglandular tissue. In contrast, the other three variants tended to produce more false-positive responses in these regions. Figure 5 further illustrates a comparison with two recent Transformer-based hybrid models, Swin-UNETR and nnFormer. The proposed method provides a closer match to the ground-truth mask and exhibits fewer false positives than either of these models.

**Fig. 6:**
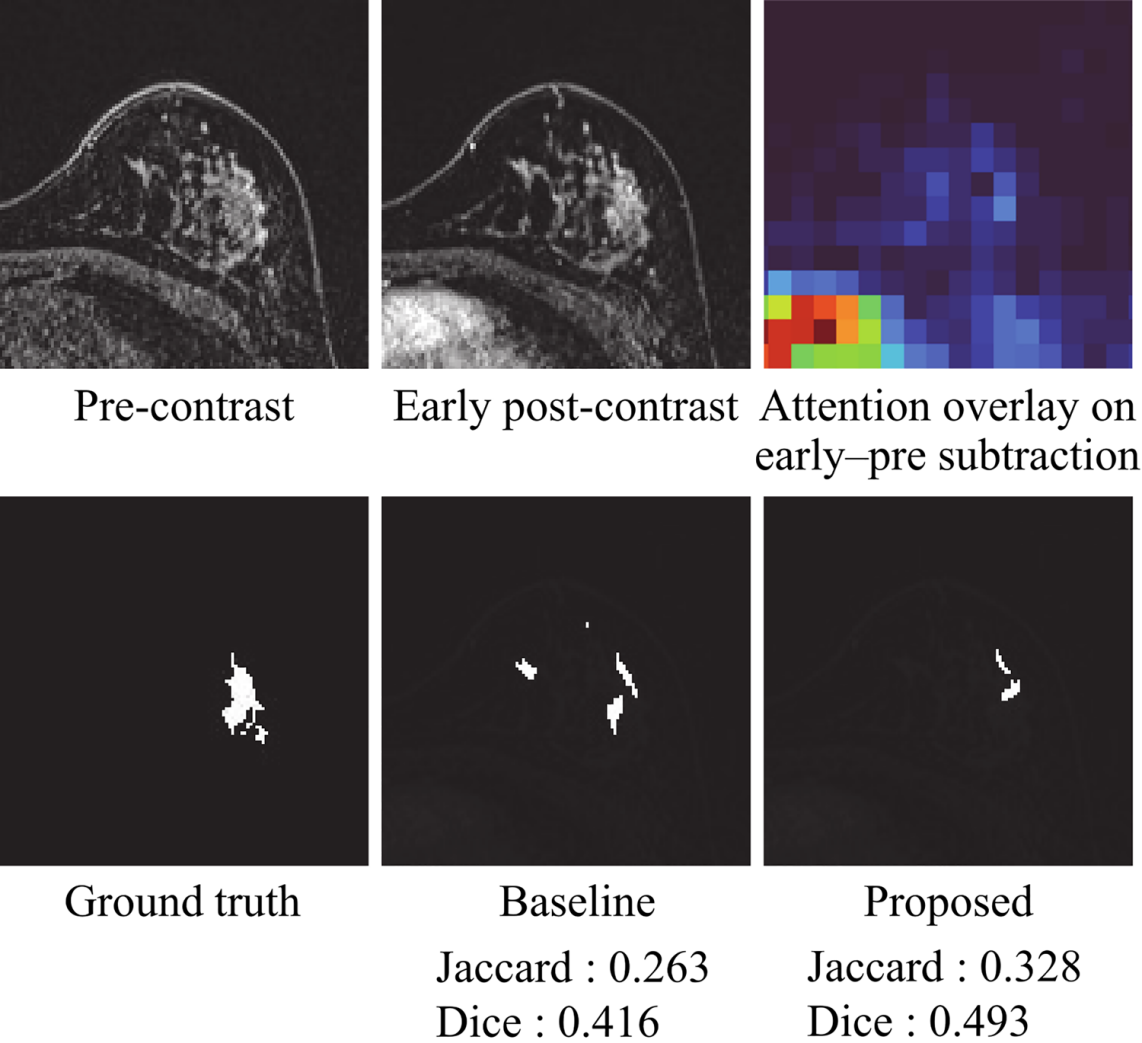
Benign case with the lowest segmentation accuracy in the test set

Table 1 summarizes the quantitative comparison among the four 3D TransUNet variants for all cases as well as for benign and malignant subgroups. For all 151 cases, the baseline 3D TransUNet, Single-Encoder + Attention, Dual-Encoder without Attention, and proposed Dual-Encoder + Temporal Attention achieved Jaccard/Dice scores of 0.585 (*p* < .001) / 0.690 (*p* < .001), 0.590 (*p* < .001) / 0.693 (*p* < .001), 0.622 (*p* < .001) / 0.723 (*p* < .001), and 0.724 / 0.802, respectively, where the *p*-values denote the results of pairwise Wilcoxon signed-rank tests versus the proposed model. All comparisons were statistically significant after Holm correction (all *p* < .05), indicating that the proposed model achieved significantly higher Jaccard and Dice scores than any of the other three ablation variants. Table 2 summarizes the quantitative comparison between the proposed method and two recent Transformer-based hybrid models. For all 151 cases, Swin-UNETR and nnFormer achieved Jaccard/Dice scores of 0.526 (*p* < .001) / 0.654 (*p* < .001) and 0.495 (*p* < .001) / 0.633 (*p* < .001), respectively, where the *p*-values indicate pairwise Wilcoxon signed-rank tests versus the proposed model. All p-values were below 0.05 after Holm correction, confirming that the proposed model significantly outperformed both Swin-UNETR and nnFormer. Across all models reported in Tables 1 and 2, the Jaccard index and Dice coefficient were consistently higher for malignant lesions than for benign lesions.

## Discussion

In this study, we developed a novel 3D TransUNet architecture that pairs two parallel CNN encoders (one for early post-contrast images and one for early–pre subtraction images) with a difference-driven temporal attention mechanism for breast DCE-MRI. This mechanism prioritizes enhancement dynamics—a hallmark of NME—to tighten boundary delineation and reduce false positives in vessels and fibroglandular tissue. To the best of our knowledge, this work is among the first to integrate difference-driven temporal attention into a hybrid 3D CNN–transformer framework for breast DCE-MRI segmentation. The proposed method not only significantly outperformed the baseline 3D TransUNet in five-fold cross-validation, but also achieved the highest Jaccard and Dice scores among all ablation variants and in comparisons with recent Transformer-based hybrid models such as Swin-UNETR and nnFormer.

As illustrated in Fig. 4, when the four 3D TransUNet variants are compared side by side, the proposed dual-encoder model using early–pre subtraction images within the temporal attention module produces the most accurate delineation and the fewest vessel-related false positives. The Single-Encoder + Attention variant showed performance comparable to the baseline, indicating that simply applying attention to a three-channel input (pre-contrast, early post-contrast, and early–pre subtraction) is insufficient to fully exploit temporal information. In contrast, the Dual-Encoder without Attention, which processes the early post-contrast and early–pre subtraction images in separate branches, yielded moderate gains over the baseline, suggesting that explicitly disentangling these two inputs helps the network extract enhancement-related features more effectively. The proposed model, which combines this dual-encoder design with the difference-driven temporal attention mechanism, achieved the largest improvement (Table 1), implying that these two components contribute in a complementary manner: features from the difference branch modulate the early-phase branch to upweight dynamically enhancing regions while suppressing background fluctuations, thereby tightening NME boundary delineation. Furthermore, Fig. 5; Table 2 demonstrate that the proposed method also outperforms recent Transformer-based hybrid models, Swin-UNETR and nnFormer, in terms of both visual quality and Jaccard/Dice scores. While these SOTA models leverage Transformer blocks to capture long-range context, they do not explicitly encode difference-driven temporal cues between phases. The superior performance and reduced false positives achieved by our method suggest that integrating a dedicated difference branch and temporal attention into a hybrid 3D CNN–Transformer architecture provides additional discriminatory power beyond that offered by existing Transformer-based segmentation frameworks.

This study is related to our previous work on NME segmentation by Hizukuri et al. [22], which used the same breast DCE-MRI dataset. In that study, region-of-interest slice images from three temporal phases (one pre-contrast and two post-contrast) were extracted and stacked to form a three-dimensional tensor, and a cross-phase convolution was applied to incorporate temporal information before feeding the resulting feature maps into a ResUNet + + encoder–decoder network. A convolutional long short-term memory layer was further introduced into the decoder to analyze slice sequences and aggregate information along the through-plane direction. Using this architecture, Hizukuri et al. achieved a mean Jaccard index of 0.563 and a mean Dice coefficient of 0.712 on the same dataset. In contrast, the current work introduces a dual-encoder 3D TransUNet with a difference-driven temporal attention mechanism and a 3D Transformer encoder that jointly model volumetric spatiotemporal patterns. As summarized in Table 1, the proposed method achieved higher Jaccard and Dice scores than the previous approach, suggesting that explicitly modeling volumetric context and difference-driven temporal enhancement can further improve NME segmentation performance beyond slice-sequence learning alone.

As shown in Tables 1 and 2, the performance improvement of the proposed method over the baseline 3D TransUNet was more pronounced for malignant than for benign lesions. This pattern likely reflects differences in vascular physiology and enhancement kinetics between lesion types. Malignant NME typically exhibits stronger, more heterogeneous early enhancement, which is attributed to increased angiogenesis and vascular permeability. Rapid wash-in and wash-out produce pronounced changes in temporal intensity that the difference-driven attention mechanism can exploit. This flexibility enabled more effective localization and delineation of malignant NME and yielded larger improvements relative to the baseline. In contrast, benign NME generally shows weaker, more homogeneous, and more gradual enhancement with persistent kinetics. Such subtle temporal cues are less salient to the attention mechanism, which prioritizes contrast-related dynamics. Consequently, the improvement was smaller for benign cases, which reflects the greater difficulty of segmenting lesions with minimal enhancement variation.

Figure 6 illustrates a benign case that yielded the lowest segmentation accuracy in the testing set. In this case, the lesion showed minimal early-phase enhancement, which resulted in suboptimal delineation by both the baseline and proposed models. Because the difference-driven temporal attention emphasizes regions with pronounced temporal changes, its effectiveness was naturally limited when such changes were absent. Notably, many low-contrast cases corresponded to benign lesions, which are clinically known to exhibit slower, milder, and more homogeneous enhancement patterns rather than the more rapid and heterogeneous enhancement typically observed in malignancies. These observations suggest that relying solely on the early post-contrast phase may be insufficient to capture the full extent of benign NME. To address this limitation, future work should consider explicitly incorporating later post-contrast phases into the model. For example, one possible extension would be to introduce a cross-attention module between the early and delayed post-contrast feature maps, allowing the network to highlight regions that remain persistently enhanced over time. Alternatively, an additional encoder could be dedicated to processing the delayed–early difference image, and the resulting feature maps could be concatenated with those obtained from the early–pre subtraction branch, so that both early and delayed temporal dynamics are jointly exploited within the temporal attention framework. Prior works have demonstrated that delayed-phase imaging provides additional diagnostic value for NME, particularly in differentiating benign from malignant lesions [29]. Capturing persistent delayed enhancement patterns characteristic of benign NME, including later phases, could improve detection and delineation of such lesions and enhance robustness across a broader spectrum of enhancement dynamics.

This study involves several limitations. First, the ground-truth NME segmentations were manually annotated by an experienced radiologist. Although this ensures clinical reliability, inter-observer variability was not assessed and may influence outcomes. Nonetheless, five-fold cross-validation showed that the proposed method achieved consistent improvements, and future multi-reader studies are warranted. Second, the dataset was relatively small, which may limit its generalizability. Expanding this research to larger and more diverse cohorts will improve the robustness and clinical applicability of the results. Third, all the imaging data were collected under a standardized MRI protocol from a single institution. Although this homogeneous setting likely contributed to the stability of the reported results, it also limits our ability to fully assess robustness to variations in scanner hardware, acquisition protocols, and contrast injection timing that are common in routine multi-center practice. Because the proposed method explicitly exploits temporal enhancement patterns, shifts in temporal sampling, sequence parameters, vendor-dependent image characteristics, or field strength may alter the learned attention patterns and potentially degrade performance if not carefully addressed. Nevertheless, subtraction (difference) imaging and relative enhancement are clinically meaningful cues in breast DCE-MRI, and our framework is designed to emphasize these relative temporal changes rather than absolute signal intensities. Therefore, we expect the proposed approach to be relatively less sensitive to certain inter-scanner and protocol-related intensity variations than methods relying primarily on absolute intensities, and the overall conclusions of the comparative analysis are likely to remain consistent. Future work will include external multi-center validation and, if needed, retraining with protocol harmonization and/or domain adaptation to further improve cross-institution generalizability. Moreover, only the pre- and early post-contrast phases were used to drive attention; incorporating delayed phases could capture persistent enhancement more accurately and improve performance for benign NME. External, multi-site validation and prospective testing are needed to ensure generalizability.

Finally, although this study focused on breast DCE-MRI, the proposed temporal attention framework is not limited to this domain. It could be extended to other contrast-enhanced applications (e.g., liver, prostate, and brain MRI) where temporal contrast dynamics are diagnostically relevant; evaluating domain adaptation and protocol harmonization will be important for such extensions.

**Table 1:**
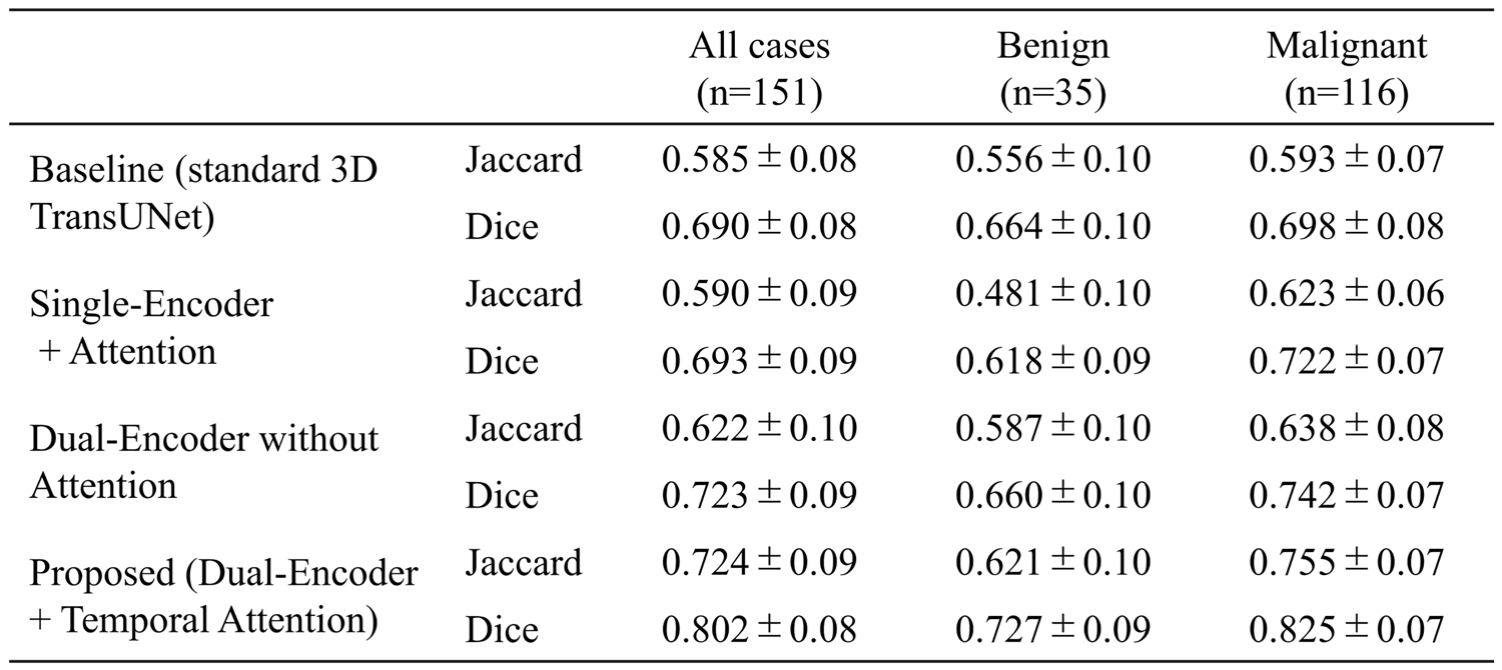
Quantitative ablation study comparing segmentation performance among the four 3D TransUNet variants: Baseline (standard 3D TransUNet), Single-Encoder + Attention, Dual-Encoder without Attention, and Proposed (Dual-Encoder + Temporal Attention), for all cases, benign lesions, and malignant lesions. Values are reported as mean ± standard deviation (SD) for the Jaccard index and Dice coefficient.

**Table 2:**
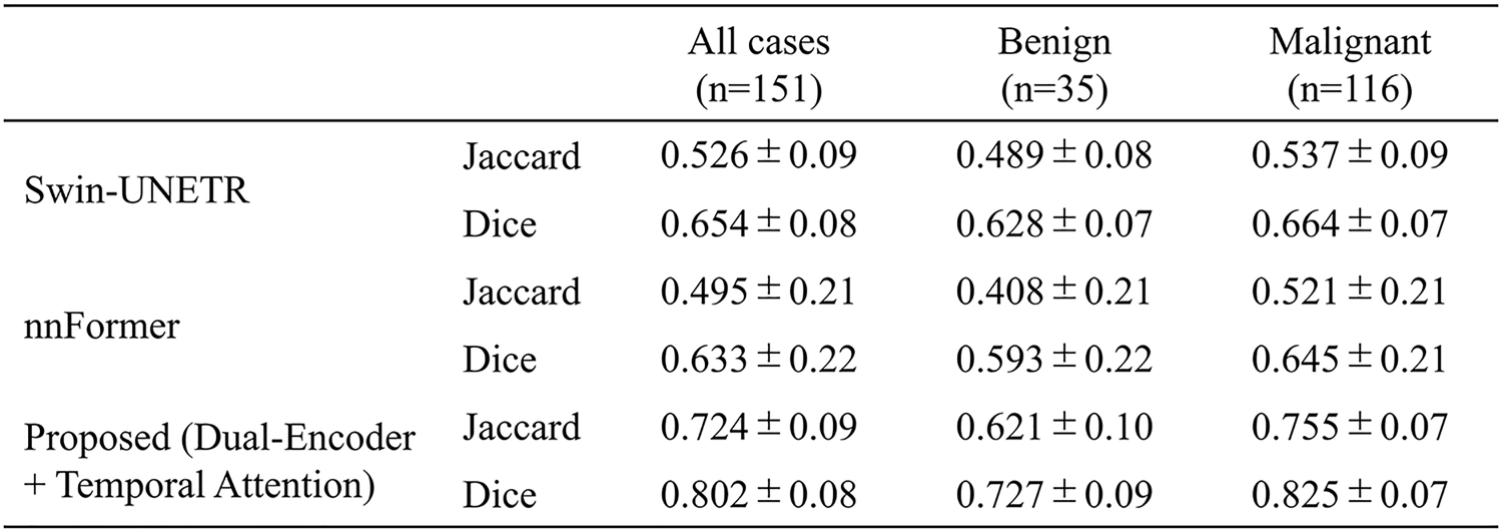
Comparison of segmentation performance among Swin-UNETR, nnFormer, and the proposed dual-encoder 3D TransUNet with difference-driven temporal attention for all cases, benign lesions, and malignant lesions. Values are reported as mean ± standard deviation (SD)

## Conclusion

In this study, we have introduced a 3D TransUNet architecture that pairs two parallel CNN encoders (for the early post-contrast and the early–pre subtraction images) with a difference-driven temporal attention module to capture dynamic changes in contrast enhancement between the pre- and early post-contrast phases of DCE-MRI. The proposed model consistently achieved higher Jaccard and Dice scores than the baseline 3D TransUNet, the three ablation variants, and recent Transformer-based hybrid models such as Swin-UNETR and nnFormer, while also providing tighter boundary delineation and fewer vessel-related false positives for NME. These results demonstrate robust NME segmentation in breast DCE-MRI and support the potential clinical utility of the proposed approach for reliable, automated assessment.

## Supplementary Information

Below is the link to the electronic supplementary material.

Supplementary Material 1

Supplementary Material 2

## Data Availability

The database used in this study is available from the corresponding author upon reasonable request.
