# Supplementary material for: Dual-encoder 3D transformer-based U-Net with temporal attention for non-mass enhancement segmentation in breast dynamic contrast-enhanced MRI
Source: Radiol Phys Technol. 2026 Jan 8;19(1):259–69. doi: 10.1007/s12194-025-01004-y (PMC12950051; doi:10.1007/s12194-025-01004-y)
Supplement: Supplementary file 1 — Supplementary Material 1 [file 12194_2025_1004_MOESM1_ESM.pdf]

Editing Certificate

This document certifies that the paper listed below has been edited to ensure that the language is clear and free of errors. The logical presentation of ideas and the structure of the paper were also checked during the editing process. The edit was performed by professional editors at Editage, a division of Cactus Communications. The intent of the author's message was not altered in any way during the editing process. The quality of the edit has been guaranteed, with the assumption that our suggested changes have been accepted and have not been further altered without the knowledge of our editors.

MANUSCRIPT TITLE

Dual-Encoder 3D TransUNet with Difference-Driven Temporal Attention for Non-Mass Enhancement Segmentation from Breast DCE-MRI

AUTHORS

Tomoki Kosugi, Ryohei Nakayama, Koji Sakai, Mariko Goto

ISSUED ON

September 23, 2025

JOB CODE

PNRJY_1

THE EDITAGE Promise QUALITY GUARANTEE

Prabh Grewal
Senior Vice President - Editage

editage | helping you get published

Since 2002, Editage has helped over 430,000 authors publish around 1.2 million research papers in scholarly journals across over 1000 disciplines through editorial, translation, transcription, and publication support services. Editage is a brand of Cactus Communications (cactusglobal.com), a science communication and technology company.

CACTUS.

GLOBAL :
+1(669) 272-1214 | request@editage.com

JAPAN :
0120-50-2987 | submissions@editage.com
