# Supplementary material for: Dual-encoder 3D transformer-based U-Net with temporal attention for non-mass enhancement segmentation in breast dynamic contrast-enhanced MRI
Source: Radiol Phys Technol. 2026 Jan 8;19(1):259–69. doi: 10.1007/s12194-025-01004-y (PMC12950051; doi:10.1007/s12194-025-01004-y)
Supplement: Supplementary file 2 — Supplementary Material 2 [file 12194_2025_1004_MOESM2_ESM.docx]

December 18, 2025

Dr. Nobuyuki Kanematsu,

Editor in Chief – Radiological Physics and Technology

Ref.: RPTE-D-25-00372

Dear Dr. Kanematsu,

Thank you very much for your letter with the reviewer’s comments of November 20, 2025, concerning our manuscript (RPTE-D-25-00372) entitled “Dual-Encoder 3D Transformer-Based U-Net with Temporal Attention for Non-Mass Enhancement Segmentation in Breast Dynamic Contrast-Enhanced MRI (revised version)” by Tomoki Kosugi, Ryohei Nakayama, Koji Sakai, and Mariko Goto.

We have carefully considered the reviewer’s comments, and then have revised the manuscript. On the separate sheets of the replies to the referee’s comments, we have provided point-by-point replies to each of the reviewer’s comments. We are sending herewith our revised manuscript (a clean copy and an annotated copy with deletions and additions) and the replies to the reviewer’s comments.

We would be grateful if the manuscript would be accepted for publication in *Radiological Physics and Technology*.

Sincerely yours,

Tomoki Kosugi

Tomoki Kosugi, B.E.

Graduate School of Science and Engineering, Ritsumeikan University

1-1-1 Noji-higashi, Kusatsu, Shiga 525-8577, Japan

Phone: +8180-7853-4372

E-mail address: ri0113vs@ed.ritsumei.ac.jp
